# Targeting macrophage Syk enhances responses to immune checkpoint blockade and radiotherapy in high-risk neuroblastoma

**DOI:** 10.3389/fimmu.2023.1148317

**Published:** 2023-06-07

**Authors:** Deepak Rohila, In Hwan Park, Timothy V. Pham, Riley Jones, Elisabette Tapia, Kevin X. Liu, Pablo Tamayo, Alice Yu, Andrew B. Sharabi, Shweta Joshi

**Affiliations:** ^1^ Division of Pediatric Hematology-Oncology, Moores Cancer Center, University of California, San Diego, San Diego, CA, United States; ^2^ Office of Cancer Genomics, University of California San Diego, San Diego, CA, United States; ^3^ Department of Radiation Medicine and Applied Sciences, Moores Cancer Center, University of California, San Diego, San Diego, CA, United States; ^4^ Department of Radiation Oncology, Dana Farber Cancer Institute, Boston, MA, United States; ^5^ Institute of Stem Cell and Translational Cancer Research, Chang Gung Memorial Hospital at Linkou, Chang Gung University, Taoyuan, Taiwan

**Keywords:** macrophage, neuroblastoma, immune suppression, Syk, T cells

## Abstract

**Background:**

Neuroblastoma (NB) is considered an immunologically cold tumor and is usually less responsive to immune checkpoint blockade (ICB). Tumor-associated macrophages (TAMs) are highly infiltrated in NB tumors and promote immune escape and resistance to ICB. Hence therapeutic strategies targeting immunosuppressive TAMs can improve responses to ICB in NB. We recently discovered that spleen tyrosine kinase (Syk) reprograms TAMs toward an immunostimulatory phenotype and enhances T-cell responses in the lung adenocarcinoma model. Here we investigated if Syk is an immune-oncology target in NB and tested whether a novel immunotherapeutic approach utilizing Syk inhibitor together with radiation and ICB could provide a durable anti-tumor immune response in an MYCN amplified murine model of NB.

**Methods:**

Myeloid Syk KO mice and syngeneic MYCN-amplified cell lines were used to elucidate the effect of myeloid Syk on the NB tumor microenvironment (TME). In addition, the effect of Syk inhibitor, R788, on anti-tumor immunity alone or in combination with anti-PDL1 mAb and radiation was also determined in murine NB models. The underlying mechanism of action of this novel therapeutic combination was also investigated.

**Results:**

Herein, we report that Syk is a marker of NB-associated macrophages and plays a crucial role in promoting immunosuppression in the NB TME. We found that the blockade of Syk in NB-bearing mice markedly impairs tumor growth. This effect is facilitated by macrophages that become immunogenic in the absence of Syk, skewing the suppressive TME towards immunostimulation and activating anti-tumor immune responses. Moreover, combining FDA-approved Syk inhibitor, R788 (fostamatinib) along with anti-PDL1 mAb provides a synergistic effect leading to complete tumor regression and durable anti-tumor immunity in mice bearing small tumors (50 mm^3^) but not larger tumors (250 mm^3^). However, combining radiation to R788 and anti-PDL1 mAb prolongs the survival of mice bearing large NB9464 tumors.

**Conclusion:**

Collectively, our findings demonstrate the central role of macrophage Syk in NB progression and demonstrate that Syk blockade can “reeducate” TAMs towards immunostimulatory phenotype, leading to enhanced T cell responses. These findings further support the clinical evaluation of fostamatinib alone or with radiation and ICB, as a novel therapeutic intervention in neuroblastoma.

## Introduction

Neuroblastoma (NB) is the most common pediatric extracranial solid tumor that arises from the sympathetic nervous system (1, 2). Patients with this disease are stratified into low, medium, and high-risk groups based on different molecular and histological parameters among which MYCN amplification is an important determinant of worse prognosis and high-risk disease (3–5). Even after extensive multimodal therapy, chances of long-term survival in the high-risk group are only 40-50% (6–9). Immune checkpoint blockade (ICB) using monoclonal antibodies directed against inhibitory T cell receptor, programmed death 1 (PD1), and its ligand, PDL1, have improved treatment regimens in various solid cancers (10). However, in high-risk NB, the outcome of these therapies is disappointing (11). Hence, novel combination therapies with ICB are needed to improve the outcomes of patients with high-risk disease.

The tumor microenvironment (TME) plays an important role in predicting responses to immunotherapy in solid tumors and our knowledge of the different components of NB TME just started emerging (12). Recently, various groups have utilized computational biology, multiplex immunohistochemistry, and sc-RNA seq approaches to define the immune landscape of NB tumors and have reported the presence of T cells, dendritic cells, B cells, myeloid cells, and natural killer cells (NK) in NB tumors (13–17). These studies have also shown that MYCN amplified (MYCN-A) tumors are immunologically cold with rare infiltration of immune cells. However, immunosuppressive myeloid cells including myeloid derived suppressor cells (MDSCs) and tumor associated macrophages are abundantly infiltrated in the MYCN-A NB tumors and are major mediators of immunosuppression in the tumor microenvironment (TME) of NB (16, 18–20). Among these myeloid cells, TAMs have recently garnered major interest as immunotherapeutic drug targets as they contribute to tumor progression and inhibition of innate and adaptive immune responses that lead to tumor immune escape and resistance to immunotherapies in NB (12, 15, 16, 19–21). Hence, “re-educating” immunosuppressive TAMs into immunogenic phenotype can improve responses to ICB or radiotherapy in high-risk NB (19, 20, 22–26).

Spleen tyrosine kinase (Syk) is a non-receptor tyrosine kinase and is mainly expressed in hematopoietic cells. Syk is an essential regulator of B cell receptor (BCR) and Fc gamma receptor(FcR)-mediated signaling (27–29) and plays a central role in the development of autoimmune diseases and in hematological malignancies such as B cell lymphomas (30). Thus, Syk inhibitors have been tested in several clinical trials for B cell malignancies, mainly chronic lymphoid leukemia (31). R788 (fostamatinib), a prodrug of active metabolite R406, is a specific Syk inhibitor that has shown great efficacy in the preclinical models of chronic lymphocyte leukemia, autoimmune diabetes, rheumatoid arthritis (32–34). R788 was also reported to reduce collagen-induced arthritis in the mouse model (33) that leads to its evaluation in phase 2 clinical trial for patients with rheumatoid arthritis (35). FDA recently approved R788 for the treatment of patients with chronic immune thrombocytopenia (36).

Recent emerging studies have shown that Syk also contributes to tumorigenesis; however, its role in the progression of solid tumors is complex (37–39). A recent report has shown that Syk acts as a tumor promoter in neuroblastoma and Syk inhibitors potentiate the effect of chemotherapeutic drugs on NB cells *in vitro* (40). However, this study did not reveal the presence of Syk in immune cells and the efficacy of Syk inhibitors in suppressing NB growth *in vivo*. Hence, mechanistic details explaining how Syk regulates immune responses in NB need to be further elucidated.

We have recently reported that Syk plays an important role in the immunosuppressive transcriptional programming of macrophages, leading to immune escape in lung adenocarcinoma (41). Herein, our objective was to evaluate the role of Syk in NB growth and anti-tumor immunity. We found that macrophage Syk is a driver of immunosuppression and neuroblastoma growth. Moreover, genetic deletion or pharmacological inhibition of Syk with R788 promotes immunostimulation, increases CD8+ T cells, and decreases tumor progression in a MYCN-driven mouse model of NB. Finally, we demonstrated that R788, in combination with anti-PDL1 mAb treatment, elicit robust anti-tumor effects in mice bearing small (50 mm^3^) NB9464 tumors and, when combined with radiation, prolonged survival of mice bearing large NB9464 tumors.

## Methods

### Human tissues and cell lines

Deidentified human neuroblastoma primary tumor samples were obtained under IRB approval (Protocol 071729) from Rady Children’s Hospital, San Diego, California, USA. NB9464 cells were cultured in Dulbecco’s modified Eagle’s medium (DMEM) containing 10% FBS and M3 base special media as described before (18, 42). Human neuroblastoma cell lines, SKNBE2, IMR32, SKNSH, SH-SY-5Y cells were cultured in DMEM supplemented with 10% FBS, 2 mM L-glutamine, 0.4 mM sodium pyruvate, non-essential amino acids and penicillin/streptomycin as described before (18). All cell lines were routinely tested for mycoplasma.

### Immunohistochemistry and immunofluorescence staining

Formalin-fixed paraffin-embedded (FFPE) tissue sections (4µm) of human NB were used for immunohistochemistry (IHC) or immunofluorescence (IF) studies. IHC and IF on paraffin-embedded human samples were performed by the UCSD Histology core facility. For IHC studies, human samples were stained with the following antibodies: anti-CD20 (ab64088, 1:100, Abcam), anti-CD68 (MA5-12407, 1:50, Invitrogen), anti-CD3 (ab16669, 1:500, Abcam) anti-CD4 (ab288724, 1:1000, Abcam), anti-CD8 (70306S, 1:50, Cell Signaling). Antigen retrieval was carried out in citrate buffer (pH 6.0, Vector Laboratories) at 95^°^C for 30 min. After antigen retrieval, tissue sections were incubated with BLOXALL (Vector Laboratories) for 10 min followed by blocking Blotto (Thermo) for 10 min. Sections were stained with primary antibodies at recommended dilutions in Blotto for 1 hr at room temperature. After washing primary antibodies, the samples were stained with anti-mouse or anti-rabbit secondary antibodies HRP Polymer (Cell IDX for 30 min at RT followed by DAB staining using DAB chromogen (VWR, 95041-478) for 5 min. The images with positive staining were captured using Olympus inverted microscope. The whole area of the tumor was selected as a field of interest, and the area with immunohistochemically positive staining within the field of interest was calculated by the Image J software after setting the thresholds. The results are expressed as the percentage of the positively immunolabelled area within the total area of the tumor. For immunofluorescence staining, antigen retrieval was carried out as described above followed by blocking with Blotto for 10 min. The tissues were then incubated with Syk (clone EP573Y, ab40781, 1:1000, Abcam) and CD68 (MA5-12407, 1:50, Invitrogen) antibodies in Blotto for 1 hr at room temperature. After two washings with 1X TBST buffer, samples were stained with anti-mouse or anti-rabbit HRP polymer-conjugated secondary antibodies (Cell IDX) for 30 min at RT. Tissues were then incubated with Alexa Fluor 488 and Alexa Fluor 594 Tyramide reagents (Thermo) for 10 min. DAPI was used to counterstain the nuclei. The images of fluorescent staining were captured using Keyence BZX-700 fluorescent microscope.

### Mouse models and therapeutic treatments

The conditional myeloid Syk knock out mouse strain (Syk^MC-KO^) and their wild type control (Syk^MC-WT^) are from the C57BL/6 background and were generated by crossing floxed Syk mice with lysozyme M (LysM) Cre recombinase transgenic mice as described previously (41). 4-6-week-old C57BL/6 mice used in these experiments were obtained from Charles River Laboratories. NB9464 (4 x 10^6^) cells were injected subcutaneously into syngeneic 4–6-week-old Syk^MC-WT^ or Syk^MC-KO^ or C57BL/6 mice. For inhibitor experiments, NB9464-bearing mice received 50 mg/kg R788, or 200 µg anti-PDL1 (clone 10F.9G2, Bio-X-cell) or isotype control LTF2 (Bio X cell) or one dose of 10Gy radiation using a SmART radiator.

### Macrophage, B cell, and T-cell depletion experiments

For macrophage-depletion experiments, mice bearing NB9464 tumors were treated with 50 mg/kg anti-CSF-1R antibody (clone AFS 98, BioXcell), administered intraperitoneally (ip) every alternate day until tumors were harvested. For B-cell depletion experiments, mice received tail vein injection of 250 µg of ULTRA-LEAF purified anti-mouse CD-20 (clone SA271G2, Bio Legend). For CD8-depletion experiments, mice bearing NB9464 tumors were treated with 200µg of anti-CD8 (clone YTS169.4) from Bio-X-Cell administered ip on day 7, 10 and 13 of tumor inoculation as described before (41).

### Single cell preparation and flow cytometric analysis

Single-cell suspensions of NB9464 tumors were prepared for flow cytometry as described before (18). Briefly, NB9464 tumors were isolated, minced, and incubated for 30-45 min at 37°C in a dissociation solution containing Hanks Balanced Salt Solution supplemented with 0.5 mg/ml collagenase IV (Sigma), 0.1 mg/ml hyaluronidase V (Sigma), 0.6 U/ml Dispase II (Roche) and 0.005 MU/ml DNAse I (Sigma). The undigested tissues were removed by passing through 70 µm nylon mesh and centrifuged at 1500 rpm for 5 min. The red blood cells were lysed using RBC lysis buffer (Pharm Lyse, BD Biosciences, San Jose, CA, USA). Myeloid cells, macrophages, and T cells were enriched using CD11b+, F4/80+, and CD90.2+ microbeads (Miltenyi Biotec, San Diego, CA) on MS columns using manufacturer’s instructions. For flow cytometry, single cells isolated from tumors were incubated with a fixable viability stain 510 (BD, Biosciences) followed by incubation with fluorescently labeled antibodies directed against mouse CD45 (30-F11; BD Biosciences), CD11b (M1/70; BD Biosciences), Gr1 (RB6-8C5; BD Biosciences), F4/80 (BM8; BD Biosciences), MHCII (AF6-120.1; BD Biosciences), PDL1 (MIH5, BD Biosciences), CD44 (IM7, BD Biosciences), CD62L (MEL-14, Biolegend), CD3 (145-2C11; eBioscience), CD4 (GK1.5,; eBioscience), CD8 (53-6.7,; eBioscience). Samples were acquired on LSR Fortessa (BD Biosciences) and analyzed withFlowJo 10 (Treestar).

### Macrophage adoptive transfer experiments

For macrophage adoptive transfer experiments, CD11b^+^ F4/80^+^ TAMs were isolated from single cell suspensions of NB9464 tumors from Syk^MC-WT^ or Syk^MC-KO^ mice by magnetic bead isolation. Purified cells were mixed 1:1 with NB9464 tumor cells, and 2 x 10^6^ total tumor cells were injected into naïve Syk^MC-WT^ or Syk^MC-KO^ mice.

### Isolation of BMDMs and hypoxia experiments

Bone marrow derived macrophages (BMDMs) were generated from 6-8 week old C57BL/6 mice as described before (43). Briefly, the bone marrow derived cells were collected by flushing the femurs and tibias of the mice with PBS using a 30-gauge needle. Following treatment with red cell lysis buffer, purified cells were cultured in RPMI + 10% FBS + 50 ng/ml MCSF (Peprotech) for 5-7 days before use. For hypoxia experiments, BMDMs were placed in a modulator incubator chamber (Billups-Rothenberg) under 1% O_2_ conditions as previously described (41, 43).

### Preparation of conditioned media and co-culture experiments

Tumor conditioned media (TCM) was prepared from confluent NB9464 cells. For this, cells were grown to 80% confluence, washed with PBS and media changed to DMEM without FBS for another 48 hrs. TCM was collected after 48 hrs. and used for co-culture experiments. BMDMs from C57BL/6 WT mice were incubated with TCM from NB9464 cells, followed by treatment with R788 for 24 hours and RNA isolation. For some experiments, BMDMs incubated with TCM from NB9464 were given hypoxia for six hrs. in the presence of 500 nM R788, followed by the preparation of nuclear extracts.

### Cell viability assays

Cell viability assay was performed on R788-treated NB9464, SKNBE2, and IMR32 cells using AlamarBlue^®^ (Roche) reagent according to manufacturer’s protocol. Briefly, 1x 10^4^ cells were seeded in 96 well plates and different concentrations of R788 were added to the culture. After 48 hours, AlamarBlue^®^ was added and plates were incubated at 37°C in 5% CO_2_ for 6 hours. Fluorescence signals were read as emission at 590 nm after excitation at 560 nm.

### Immunoblotting

CD45- tumor cells, CD45+ immune cells, CD11b+F4/80+ TAMs from NB9464 tumors, BMDMs, CD90.2 + T cells, CD19+ B cells isolated from splenocytes of NB9464-bearing mice and *in vitro* cultured SKNBE2, IMR32, SKNBE2, SH-SY-5Y and NB9464 cells were solubilized in RIPA buffer containing protease and phosphatase inhibitors. Proteins were run on SDS-PAGE gels, electro transferred to nitrocellulose membranes and were immunoblotted with antibodies from Syk (Santa Cruz), β-actin (Santa Cruz).

### qPCR analysis

Total RNA was isolated from BMDMs or TAMs from NB9464 tumors or bulk NB9464 tumors using the Qiagen RNAeasy kit (Qiagen, Hilden, Germany), and cDNA was synthesized using iscript cDNA synthesis kit (Bio-Rad, Hercules, CA) according to manufacturer’s instructions. SYBR green-based qPCR was performed using murine primers to immune-responsive genes as described earlier (41). The data were quantified with the comparative Ct method for relative gene expression. GAPDH was used as the housekeeping gene.

### RNA-sequencing

RNA was harvested from WT BMDMs incubated with NB9464 TCM and TAMs isolated from Syk^MC-WT^ and Syk^MC-KO^ mice bearing NB9464 tumors, and R788-treated NB9464 tumors using Qiagen RNAeasy kit (Qiagen, Hilden, Germany). RNA libraries were prepared and sequenced using standard Illumina protocols. RNA sequencing was performed by Novagene Corporation Inc (Sacremento, CA). mRNA profiles were generated by single read deep sequencing, in triplicate, using Illumina HiSeq2000. FASTQ files from sequencing experiments were mapped to mouse genome build mm10 using STAR (44) with default parameters, with reads quantified by Salmon (45) in a standard BCBio RNAseq pipeline. Differential expression analysis was then performed using the DESeq2 R package (46). Apeglm was used for empirical shrinkage effect size estimation (47). P-values were multiple hypothesis corrected using the Benjamini-Hochberg method in the python stats models package (48). All heatmaps showing relative expression of selected genes were created using the seaborn python package (49) on the Z-scores of the TPM-normalized expression.

### Sc-RNA-seq data acquisition, integration, and analyses

Single cell RNA-seq data from TH-MYCN murine tumors (n = 3) were downloaded from accession GSE180101 and analyzed in the R package Seurat. First, anchor genes were used to integrate and normalize all cells across all mice into a single data set that could be clustered and visualized as a UMAP, using default settings. Next, the CIPR package, combined with canonical marker genes in cases where the cluster identity was ambiguous, was used to assign cluster identities with the mmrnaseq reference. All clusters identified as non-immune cells were grouped together as “Tumor” for clarity. Finally, a violin plot of Syk expression across different cell types was generated.

### Statistical analysis

Data is represented as mean ± SEM. Differences in survival were determined using Kaplan-Meier method and using the log-rank test. Statistical significance was determined by parametric or nonparametric student’s t-test when only two groups were compared or by one-way ANOVA with Tukey’s *post-hoc* testing for multiple pairwise testing with more than two groups. In all cases, * p < 0.05, ** p < 0.01 *** p < 0.001, **** p < 0.0001, ns = not significant. Statistical analysis was performed using GraphPad Prism V.9 software.

## Results

### High-risk MYCN-amplified tumors are ‘immunologically cold’ tumors with abundant infiltration of immunosuppressive macrophages

**Figure 1:**
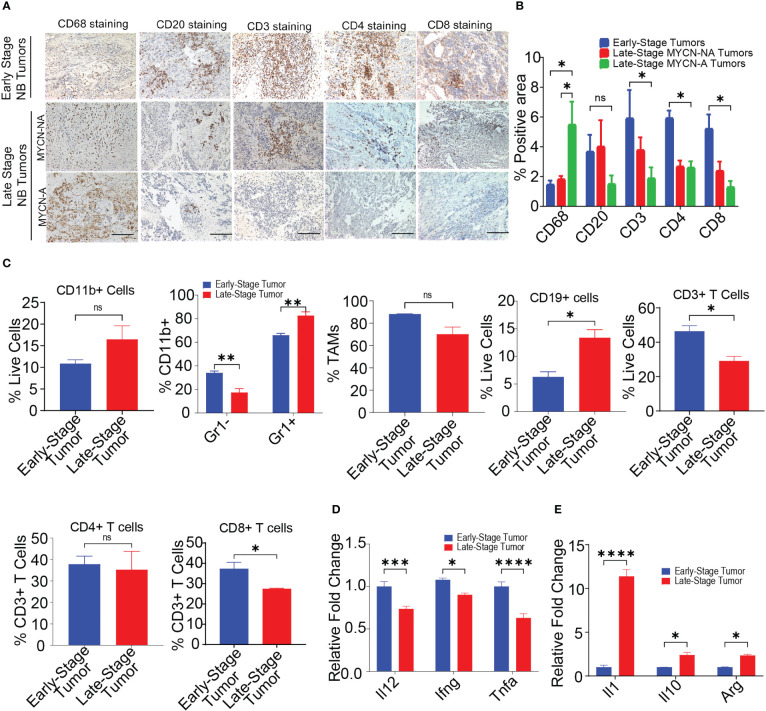
MYCN amplified tumors are abundantly infiltrated with immunosuppressive macrophages. **(A, B)** Representative images **(A)** and quantification of positive IHC staining **(B)** for CD68, CD20, CD3, CD4, and CD8 in tissue sections from early-stage MYCN-NA (n = 5), late-stage MYCN-NA (n= 7) and MYCN-A (n = 6) human NB; scale bar = 50μm. **(C)** The percentages of intratumoral CD11b+ myeloid cells, CD11b+Gr1- monocytes and CD11b+Gr1+ granulocytes, CD3+ T cells, CD4+ T cells, and CD8+ T cells in early-stage and late-stage murine NB9464 tumors (n = 4). **(D, E)** Relative mRNA expression of immunostimulatory **(D)** and immunosuppressive genes **(E)** in TAMs isolated from early-stage and late-stage NB9464 tumors (n =3). * p < 0.05, ** p < 0.01 *** p < 0.001, **** p < 0.0001, ns, not significant.

The immune microenvironment of NB plays an important role in disease progression and predicting responses to immunotherapy (12, 50). However, limited studies are available which exemplify the presence of immune cells in NB tumors (13, 15, 16). To delineate the immune composition of NB tumors, we performed IHC on 18 human patient samples {n = 5, early-stage NB; n = 7, non-MYCN amplified (MYCN-NA) late-stage NB, n = 6, MYCN amplified (MYCN-A) late-stage NB} collected under IRB protocol (071729) (**Supplementary Table S1**). IHC on human NB patient samples reveals the higher infiltration of CD20+ B cells, CD3+ T cells, CD4+ T cells, and CD8+ T cells in early-stage tumors, followed by decreased or rare infiltration of these cells in late-stage MYCN-NA tumors and MYCN-A tumors respectively (**Figures 1A, B**). Interestingly, we found increased infiltration of CD68+ macrophages in late-stage MYCN-A tumors compared to early-stage NB and late-stage MYCN-NA tumors (**Figures 1A, B**).

We implanted murine MYCN-A (NB9464) cells in C57BL/6 mice to further study these dynamics using *in vivo* models. To evaluate the immune composition of these tumors, we used early-stage tumors (tumor volume = 100-200 mm^3^) and late-stage tumors (tumor volume = 800-1000mm^3^) and evaluated the immune infiltrates using flow cytometry. Like human tumors, late-stage murine MYCN-A tumors also showed decreased infiltration of CD3+ T cells, and CD8+ T cells (**Figures 1C**, **S1**, **S2**). Interestingly, we found higher infiltration of CD11b+ Gr1+ granulocytes and CD19+ B cells in late-stage tumors with no changes in the infiltration of CD11b^+^Gr1^-^F4/80^+^ TAMs (**Figures 1C**, **S2**). Considering the results from both human and murine tumors, we found that late-stage MYCN-A tumors showed high infiltration of macrophages and B cells. Hence, we investigated if the deletion of either of these cell types can impede NB tumor progression. For this, NB9464 cells were subcutaneously implanted into C57BL/6 mice and were treated with macrophage-depleting anti-CSF1R antibodies (clone AFS98) or B-cell depleting ULTRA-LEAF™ purified anti-CD20 mAb (SA271G2, Biolegend). We found that administration of anti-mouse CSF1R depleted macrophages from the treated mice and suppressed tumor growth in mice implanted with NB9464 tumors (**Figures S3A–C**). However, administration of ULTRA-LEAF™ purified mAb depleted B cells, but did not suppress NB9464 tumor growth (**Figures S3A–C**). Taken together, these results suggest that macrophages and not B cells play an important role in the progression of NB, and these results were also validated by other groups (19, 51).

Several studies have shown that macrophages promote tumor growth by facilitating immunosuppression in NB (52, 53). Verhoeven et al, have recently shown that macrophages with an immunosuppressive ‘M2’ phenotype significantly correlated with decreased survival, while monocytes and macrophages with an immunostimulatory ‘M1’ phenotype significantly correlated with increased survival (15). Similarly we found that TAMs isolated from late stage murine MYCN-A NB tumors exhibited decreased expression of proinflammatory genes like *Il12*, *Ifng*, and *Tnfa* and increased expression of immunosuppressive genes like *Il1*, *Il10*, and *Arg* (**Figures 1D, E**). Altogether, these results suggest that immunosuppressive macrophages predominate in late-stage MYCN-A NB tumors, and approaches that aim to polarize macrophages into immunostimulatory phenotype can be used as an effective strategy to treat high-risk NB.

### Macrophage Syk fosters neuroblastoma growth

**Figure 2:**
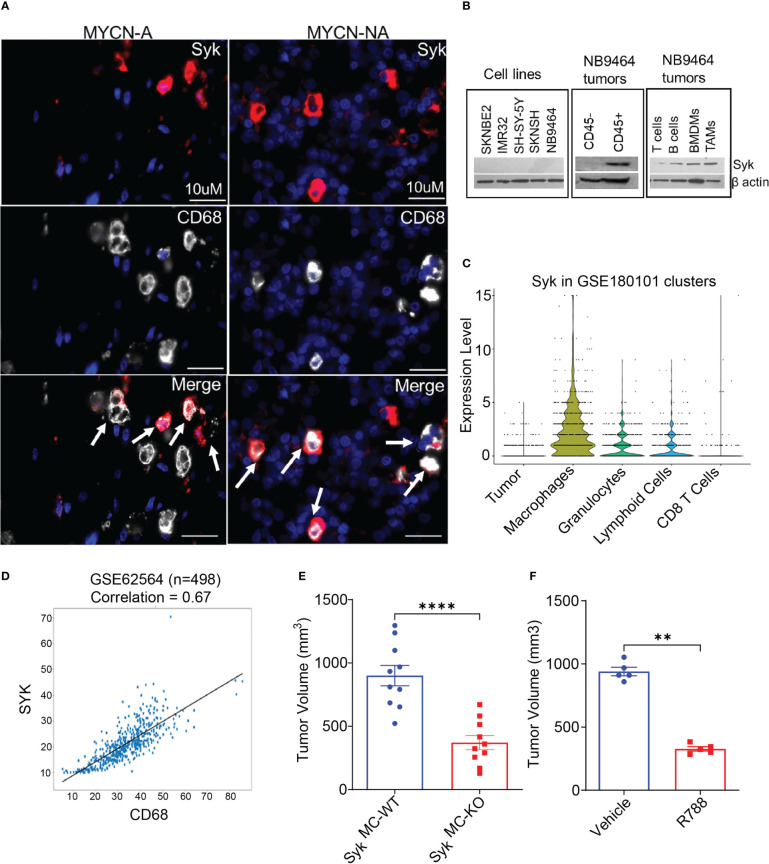
Macrophage Syk promotes neuroblastoma growth. **(A)** Figure shows IF staining of SYK and CD68 in human MYCN-A and MYCN-NA NB tissue sections. The tissue sections were stained with DAPI to detect nuclei. The cells stained positive for SYK, and CD68 are shown in the merged figure; scale bar = 10μm. **(B)** Protein expression of Syk in human NB cell lines: SKNBE2, IMR32, SH-SY-5Y and SKN-SH; murine NB9464 cells, CD45- tumor cells, CD45+ immune cells, CD11b+F4/80+ TAMs isolated from NB9464 tumors, and CD19+ B cells, CD90.2+ T cells, BMDMs isolated from splenocytes of NB9464-bearing mice. **(C)** Syk expression in tumor cells and different immune cells in spontaneous TH-MYCN murine tumors, using the publicly available single cell RNAseq data set (GSE180101). **(D)** Correlation between SYK and CD68 was analyzed using a dataset containing 498 NB samples (Cohort SEQC, GSE62564). **(E)** Tumor volume of NB9464 tumors implanted in Syk^MC-WT^ and Syk^MC-KO^ mice (n=10), ****p≤ 0.0001. **(F)** Tumor volume of NB9464 tumors treated with 50mg/kg R788 (five times a week) (n = 5), **p≤ 0.01.

We have recently shown that Syk, a macrophage key kinase, promotes immunosuppression and tumor growth in different syngeneic murine tumor models (41). As macrophages predominate in the NB TME, we asked if Syk is expressed in the NB tumors. To study the accumulation of Syk-positive macrophages in NB, we immunostained human NB patient tissues with SYK and macrophage marker CD68. Immunofluorescence microscopy revealed the presence of SYK+ cells in the tissue sections of both MYCN-A and MYCN-NA NB human specimens and immunoreactivity of SYK in CD68+ TAMs (**Figure 2A**). A recent study has shown that Syk is abundantly present in both MYCN-A and MYCN-NA human NB tumors, but it is expressed explicitly in human MYCN-NA NB cell lines (40). Hence, we evaluated the protein expression of Syk in MYCN-A (SKNBE2, IMR 32) and MYCN-NA (SH-SY-5Y, SKNSH) human NB cell lines and immune cells isolated from the mice bearing NB9464 (MYCN-A) tumors. Contrary to a previous report (40), we did not find the expression of Syk in MYCN-A or MYCN-NA human NB cell lines or murine NB9464 cells (**Figure 2B**). We found that Syk is specifically expressed in the CD45+ immune cells isolated from the murine NB9464 (MYCN-A) tumors and not in CD45-tumor cells (**Figure 2B**). Most notably, we found that Syk is specifically expressed in CD11b+F4/80+ TAMs isolated from NB9464 tumors; BMDMs and CD19+ B cells isolated from splenocytes of mice bearing NB9464 tumors (**Figure 2B**). We found minimal expression of Syk in CD90.2+ T cells isolated from splenocytes of mice bearing NB9464 tumors (**Figure 2B**). We next analyzed the Syk expression in tumor cells and different immune cells in spontaneous TH-MYCN murine tumors, using the publicly available single cell RNAseq data set (GSE180101). We found that Syk is maximally expressed in macrophages, followed by granulocytes and lymphoid cells, but not in tumor cells and CD8+T cells (**Figure 2C**). Most notably, SYK expression is also positively correlated with expression of macrophage marker CD68 in a publicly available GEO dataset (GSE62564) (**Figure 2D**).

As Syk is present in both B cells and macrophages, and deletion of B cells has no impact on NB growth (**Figure S3A**), we hypothesized that Syk is a marker of neuroblastoma-associated macrophages and might have a functional role in NB growth. To study the contribution of macrophage Syk on immunosuppression and NB tumorigenesis, we studied the growth of subcutaneous NB9464 tumors in conditional myeloid Syk KO mice model, generated as described before (41). We observed that tumor growth of NB9464 was reproducibly and significantly reduced in Syk^MC-KO^ animals as compared to that in Syk^MC-WT^ animals (**Figure 2E**). We next asked if pharmacological inhibition of Syk can reduce NB growth. Like genetic deletion, pharmacological inhibition of Syk with FDA approved Syk inhibitor, Fostamatinib or R788 also reduced tumor growth in WT mice implanted with NB9464 tumors (**Figure 2F**). Most notably, we found that Syk inhibition didn’t directly affect the survival of MYCN-A NB cells *in vitro* (**Figure S4**).

### Syk blockade can overcome NB-related immunosuppression and enhances T cell activation *in situ*

**Figure 3:**
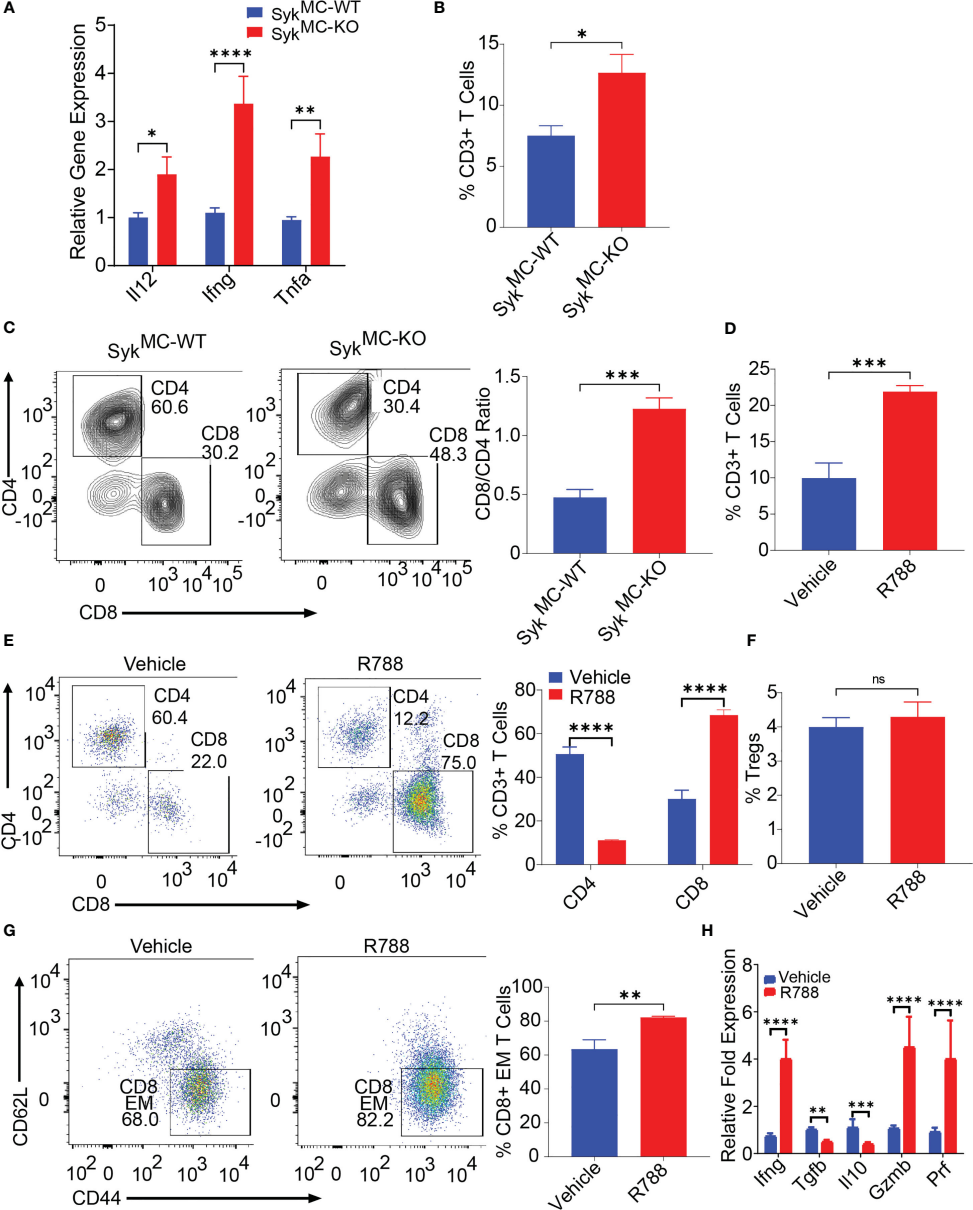
Syk blockade can overcome NB-related immunosuppression and enhances T cell infiltration and activation *in situ*. **(A)** mRNA expression of *Il12*, *Ifng*, *Tnfa* in Syk^MC-WT^ and Syk^MC-KO^ NB9464 tumors. **(B, C)** The percentages of intratumoral CD3+ T cells **(B)** and CD4+ T cells and CD8+ T cells **(C**, left panel shows FACS plots and right panel shows CD8/CD4 ratio) in Syk^MC-WT^ and Syk^MC-KO^ NB9464 tumors. **(D–G)** FACS quantification of CD3+ T cells **(D)** CD4+, and CD8+ T cells **(E**, left panel shows FACS plots and right panel shows quantification), CD4+CD25+FOXP3+ T regs (gated on CD4+ T cells) **(F)**, and CD44+CD62L- effector T cells (gated on CD8+ T cells (**G**, left panel shows FACS plots and right panel shows quantification) in R788 treated NB9464 tumors). **(H)** Relative mRNA expression of *Ifng*, *Il10, Tgfb*, *Gzm* and *Prf* in R788-treated NB9464 tumors. ns, not significant. *p < 0.05, ** p < 0.01 *** p < 0.001, **** p < 0.0001.

Since inhibition of myeloid Syk was protective against NB, we asked if blockade of Syk can overcome NB-induced immunosuppression. To explore how Syk blockade affected the immune responses in the tumor- bearing Syk^MC-WT^ and Syk^MC-KO^ mice, we analyzed immune response genes in these mice. We found elevated expression levels of proinflammatory mRNAs like *Il12*, *Ifng*, *Tnfa* in the tumors isolated from Syk^MC-KO^ mice as compared to Syk^MC-WT^ mice (**Figure 3A**). As Syk deletion, enhanced immune stimulation in the TME, we next analyzed if Syk blockade has any impact on the infiltration and activation of T cells. We found that Syk blockade stimulated T cell recruitment into NB tumors as CD3+ and CD8+ T cell content was significantly increased in the tumors implanted in Syk^MC-KO^ mice and in NB9464 tumors treated with R788 (**Figures 3B–E**). We found a significant decrease in the relative infiltration of CD4+ T cells in the R788-treated tumors (**Figure 3E**). Hence, we analyzed if R788 has any impact on Treg population in the NB tumors. We did not find any changes in the CD4^+^CD25^+^FoxP3^+^ Tregs in the vehicle and R788-treated tumors (**Figure 3F**). However, in addition to an increase in CD8 T cell frequency, NB-infiltrating CD8+ T cells were markedly activated with a CD44^+^CD62L^-^ effector CD8+ T cells are highly infiltrated in R788-treated tumors (**Figure 3G**). Moreover, we also found high expression of the T_H_1 cytokine *Ifng*, and decreased expression of immunosuppressive cytokines, *Il10* and *Tgfb* in CD90.2+ T cells isolated from R788 treated tumors (**Figure 3H**). Interestingly, consistent with a cytotoxic effector phenotype, the expression of *Gzm* and *Prf* was also upregulated in the T cells isolated from the R788-treated tumors (**Figure 3H**). Remarkably, Syk inhibition did not directly activate T cells as neither Syk deletion nor treatment of T cells with R788 affected T cell proliferation *ex vivo* (**Figures S5A, B**). Collectively these data suggest that targeting Syk can overcome NB-related immunosuppression and enhances cytotoxic CD8+ T cell responses in NB.

### Syk inhibition induces immunostimulatory reprogramming of intratumoral macrophages

**Figure 4:**
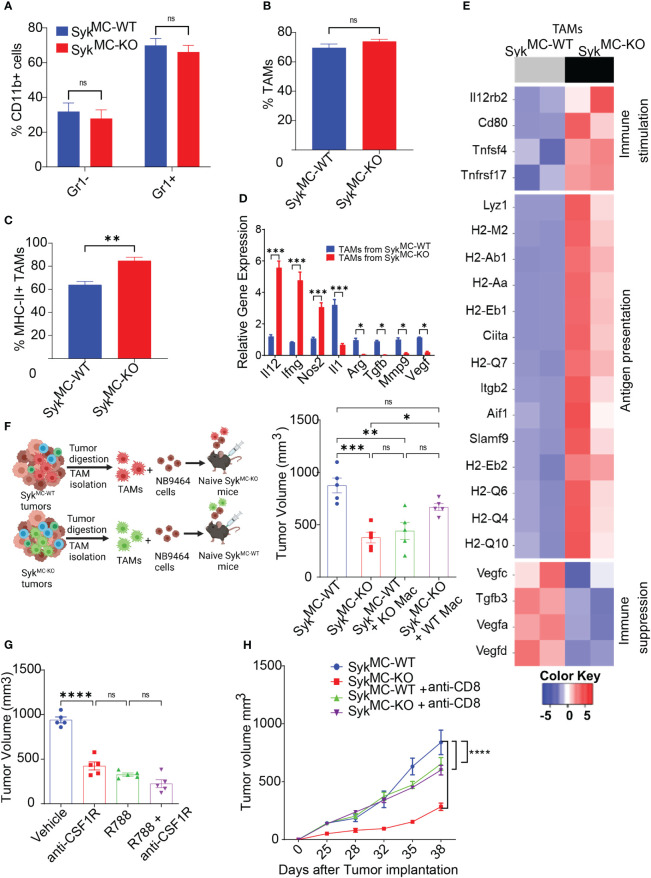
Syk inhibition induces immunostimulatory reprogramming of intratumoral macrophages. **(A–C)** The percentages of intratumoral CD11b+Gr1-, CD11b+Gr1+ cells **(A)**, TAMs **(B)**, and MHCII+ TAMs **(C)** in Syk^MC-WT^ and Syk^MC-KO^ NB9464 tumors. **(D)** Relative mRNA expression of immune response genes in TAMs isolated from Syk^MC-WT^ and Syk^MC-KO^ NB9464 tumors. **(E)** Relative mRNA expression of immune response genes in TAMs isolated from Syk^MC-WT^ (grey) and Syk^MC-KO^ (black) NB9464 tumors as determined by RNA sequencing. **(F)** Left panel shows schema of adoptive transfer experiments. Right panel shows tumor volumes of Syk^MC-WT^ and Syk^MC-KO^ NB9464 tumors adoptively transferred with Syk^MC-WT^ and Syk^MC-KO^ TAMs. **(G)** NB9464 tumor volumes of C57BL/6 mice treated with anti-CSF1R mAb and/or R788. **(H)**. NB9464 tumor volumes from Syk^MC-WT^ and Syk^MC-KO^ mice treated with anti-CD8 or isotype control antibodies (n = 5). ns, not significant. * p < 0.05, ** p < 0.01 *** p < 0.001, **** p < 0.0001.

Since Syk deletion promoted recruitment and activation of T cells in NB tumors with no impact on T cell proliferation *ex vivo* (**Figure S5**), we hypothesized that myeloid Syk deletion reprograms macrophages to promote immunostimulatory responses *in vivo*. We observed no changes in the infiltration of CD11b^+^Gr1^-^ monocytes, CD11b^+^Gr1^+^ granulocytes, and CD11b^+^Gr1^-^F4/80^+^ TAMs in Syk^MC-WT^ and Syk^MC-KO^ tumors (**Figures 4A, B**). Whereas genetic deletion of Syk didn’t alter the accumulation of TAMs in the tumors, it enhanced the expression of MHCII+ TAMs in Syk^MC-KO^ tumors (**Figure 4C**). In addition, Syk enhanced the expression of immunosuppressive cytokines like *Arg*, *Tgfb*, *Il1*, *Mmp9*, and *Vegf* in TAMs and inhibited expression of pro-inflammatory genes, *Il12*, *Ifng*, and *Nos2* (**Figure 4D**). These results were also validated by RNA-seq on TAMs from NB9464 tumors which also revealed high expression of genes related to antigen presentation and immune stimulation in Syk^MC-KO^ mice (**Figure 4E**). These results indicate that Syk skews the macrophages towards an immunosuppressive phenotype in NB tumors.

To confirm that macrophage Syk controls tumor growth and inhibits adaptive immune responses in NB, TAMs isolated from Syk^MC-WT^ and Syk^MC-KO^ were mixed in a 1:1 ratio with NB9464 tumor cells and were adoptively transferred into different Syk^MC-WT^ and Syk^MC-KO^ mice (**Figure 4F**). The adoptive transfer of WT macrophages into Syk^MC-KO^ mice showed an increase in tumor growth. In contrast, the transfer of Syk KO macrophages suppressed tumor growth in Syk^MC-WT^ mice (**Figure 4F**). Similarly, treatment of mice bearing NB9464 tumors with R788 or macrophage depleting anti-CSF1R antibody showed that anti-CSF1R ab or R788 treatment alone significantly blocked tumor growth, but together did not show additive effects on tumor regression (**Figure 4G**). Most notably, we found that Syk inhibition did not reduce tumor growth in CD8-depleted mice suggesting that Syk inhibition reduces tumor growth by promoting immunostimulatory transcriptional programming in macrophages leading to the recruitment and activation of CD8+ T cells (**Figure 4H**). These results implicate that Syk blockade remodels immune microenvironment towards immunostimulation, leading to enhanced CD8+ T cell responses in NB.

### Syk signaling promotes stabilization of HIF1 alpha to control macrophage polarization

**Figure 5:**
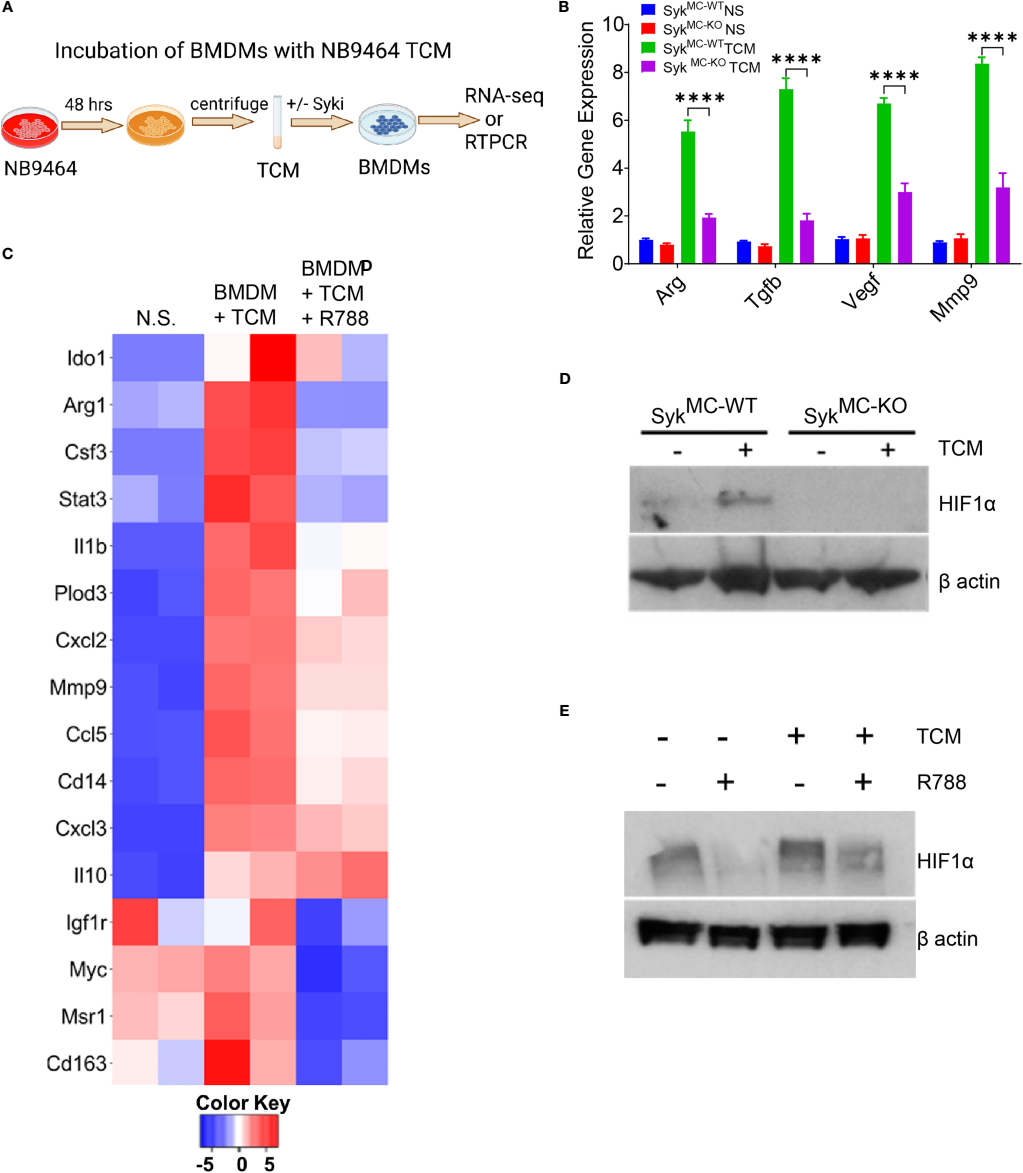
Syk signaling promotes stabilization of HIF1 alpha to control macrophage polarization. **(A)** Schema showing TCM experimental details. **(B)** mRNA expression of immunosuppressive genes in BMDMs incubated with NB9464 TCM. **** p< 0.0001. **(C)** Relative mRNA expression of genes related to immune suppression in BMDMs incubated with NB9464 TCM and treated with 500 nM R788 as determined by RNA sequencing. **(D, E)** Western blot analysis of nuclear extracts for HIF1α from Syk^MC-WT^ and Syk^MC-KO^ BMDMs incubated with NB9464 TCM **(D)** or WT BMDMs incubated with NB9464 TCM and treated with 500 nM R788 **(E)** and exposed to hypoxic conditions (1% O_2_).

Since Syk induces polarization of macrophages into immunosuppressive phenotype *in vivo*, we investigated if Syk promotes transcriptional programming of macrophages *in vitro*. For this, we incubated BMDMs derived from Syk^MC-WT^ and Syk^MC-KO^ or C57BL6 WT mice with tumor-conditioned media (TCM) from NB9464 cells as shown in the schema (**Figure 5A**). We found that stimulation of Syk^MC-WT^ and Syk^MC-WT^ BMDMs with NB9464 TCM increases polarization of macrophages in immunosuppressive phenotype only in Syk^MC-WT^ BMDMs (**Figure 5B**). RNA-seq data revealed that incubation of WT BMDMs with NB9464 TCM enhanced the expression of immunosuppressive genes like *Ido1*, *Arg1*, *Il1b*, *Mmp9*, *Il10*, and treatment with R788 significantly decreased the expression of these genes (**Figure 5C**).

Next, we investigated the mechanism by which Syk regulates macrophage immune responses in neuroblastoma tumors. We have previously shown that Syk controls the stabilization of HIF1α to promote tumor growth and immunosuppression (41). We found that BMDMs from Syk^MC-WT^ mice, when exposed to NB9464 TCM and hypoxic conditions (1% O_2_) showed higher stabilization of HIF1α in Syk^MC-WT^ BMDMs only and not in Syk^MC-KO^ BMDMs (**Figure 5D**). Similarly, WT BMDMs incubated with NB9464 TCM and exposed to hypoxia showed higher stabilization of HIF1α and R788 destabilized HIF1α in these BMDMs (**Figure 5E**). Hence, these results suggest that Syk controls stabilization of HIF1α in macrophages to promote immunosuppression in MYCN-A tumors.

### Syk inhibition with immune checkpoint blockade is effective in small NB9464 tumors

**Figure 6:**
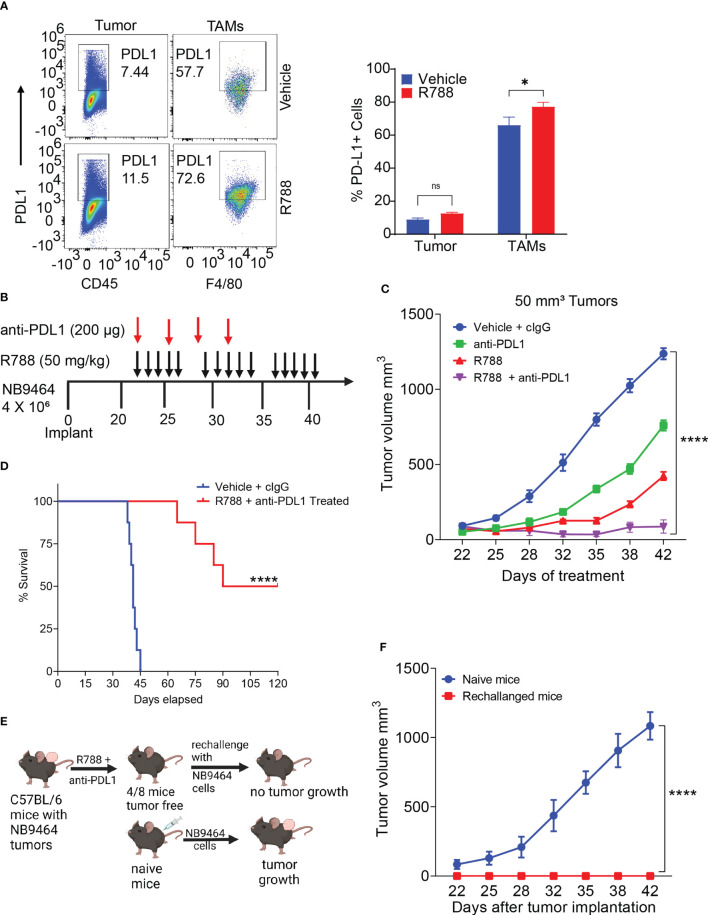
Syk inhibition synergizes with checkpoint blockade to enhance anti-tumor immune responses in small neuroblastoma tumors. **(A)** Select pseudo-color plots (left panel) and quantification (right panel) of PDL1 in CD45- tumor cells and CD11b+F4/80+ TAMs from vehicle and R788-treated NB9464 tumors. ns, not significant. *p < 0.05. **(B)** NB9464 tumors were implanted into C57 BL/6 mice and treated with different inhibitors according to the depicted schema. **(C)** Tumor volume of NB9464 tumors implanted in C57BL/6 WT mice treated with either R788 or PDL1 or in combination, as shown in **(B)**. **(D)** Kaplan Meir survival curves of mice treated with R788 and anti-PDL1 mAb. **(E)** Schema showing the strategy to rechallenge the cured mice. **(F)** Tumor volume of NB9464 tumors implanted in naïve C57BL/6 mice (n = 6) and cured mice (n = 4) from Figure **(C)** **** p < 0.0001.

Unlike some adult cancers, most pediatric cancers are immunologically cold and resistant to ICB. As Syk inhibition has remodeled the TME towards immunostimulation, we next determined if Syk inhibition combined with ICB can improve survival outcomes in NB. In line with previous report (54), we found that CD45- tumor cells exhibited low expression of PDL1 (**Figures 6A**, **S6**). Interestingly, we found that PDL1 was expressed on CD11b+F4/80+ TAMs isolated from NB9464 tumors and the expression of PDL1 further increased on R788 treatment (**Figure 6A**). Hence, we hypothesized that combining anti-PDL1 mAb with R788 can improve anti-tumor immune responses in neuroblastoma. We found that the combination of R788 and anti-PDL1 blockade provided complete tumor regression in 50% (4/8) of the mice (**Figures 6B, C**) and exhibited disease-free survival past 80 days (**Figure 6D**). None of the control untreated mice survived after day 45 (**Figure 6D**). Four mice which were disease-free after treatment were rechallenged with NB9464 tumors after three months following the completion of the last dose of treatment, and 100% (4/4) of mice rejected the tumor growth, and none of these mice developed tumor growth (**Figures 6E, F**). In contrast, all 6/6 naïve mice challenged in parallel with NB9464 cells showed progressive tumor growth (**Figure 6F**). These results suggest that Syk inhibitor sensitized NB tumors to ICB and cured the mice bearing small NB9464 tumors.

### Radiation combined with Syk inhibition and checkpoint blockade is effective in big NB9464 tumors

**Figure 7:**
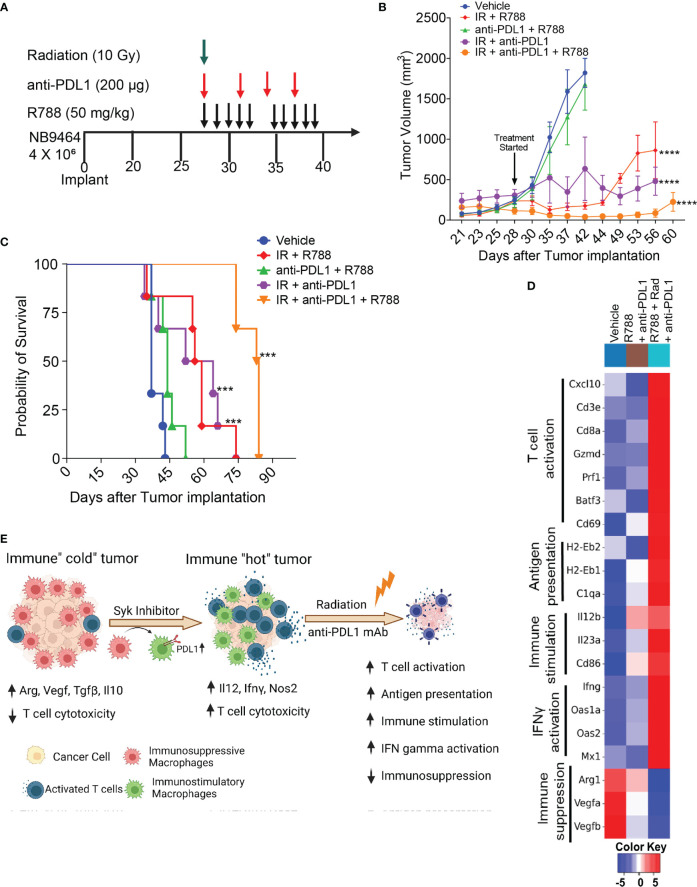
Radiation combined with R788 and anti-PDL1 mAb improved survival in mice bearing large NB9464 tumors. **(A)** NB9464 tumors implanted in C57BL/6 mice and treated according to the depicted schema. **(B)** Tumor volume of NB9464 tumors treated as mentioned in **(A)**, **** p < 0.0001. **(C)** Kaplan Meir survival curves of mice treated with the combination of R788 or anti-PDL1 mAb or radiation. *** p < 0.001. **(D)** Heat map of immune-related mRNA expression in bulk NB9464 tumors treated with R788, anti-PDL1 mAb and radiation. **(E)** Graphical abstract showing that R788 can turn immune “cold” tumors into "hot" inflamed tumors, thus enhancing the efficacy of anti-PDL1 blockade and radiation in high-risk NB.

Our results showed that R788, together with anti-PDL1 mAb, cured mice bearing small NB9464 tumors (**Figure 6**). Since, most of the neuroblastoma patients had metastatic disease at the time of diagnosis, we investigated if R788 and anti-PDL1 mAb could cure mice when treatment was initiated at later stage when the tumor volume reached 250 mm^3^. This treatment regimen was ineffective at a later-stage, and none of the treated mice was tumor-free (**Figures 7A–C**). Since, radiation therapy is an important modality in frontline treatment of high-risk NB (55) and previous studies have shown that radiation can enhance the efficacy of ICB in solid tumors (56, 57) by enhancing T cell infiltration in the TME and boosting IFN-gamma mediated immune responses (58–60), we evaluated if combined treatment of R788 along with radiation and anti-PDL1 can provide complete regression in large NB9464 tumors. We found that radiation together with anti-PDL1 or R788 was effective in regressing tumor growth, but the combination of R788 together with anti-PDL1 and radiation significantly decreased tumor burden and prolonged overall survival of mice bearing big NB tumors (**Figures 7B, C**). The median survival time of different treatment groups were: vehicle mice: 37 days, R788 + anti-PDL1 mAb: 44 days, Rad + R788: 57.5 days, Rad + PDL1: 58 days, and Rad + R788 + anti-PDL1: 84 days (**Figures 7B, C**). RNA-seq data also shows that Rad + R788 + anti-PDL1 treated NB9464 tumors showed increased expression of genes related to antigen presentation, T cell activation, immune stimulation, IFN gamma activation and decreased expression of immunosuppressive genes as compared to Vehicle, R788 + anti-PDL1 mAb treated tumors (**Figure 7D**). Taken together, these results suggest that R788 has a capacity to remodel the immune “cold” tumors into immune “hot” tumors by reprogramming the macrophages and together with anti-PDL1mAb and radiation it can regress tumor growth in high-risk MYCN-A NB (**Figure 7E**).

## Discussion

Tumor associated macrophages (TAMs) are abundantly infiltrated in human and murine NB tumors and play an instrumental role in NB progression and impeding responses to ICB (15, 19, 20). Based on our previous findings that macrophage Syk promotes immunosuppression in lung adenocarcinoma (41), herein, we investigated the functional significance of macrophage Syk in neuroblastoma tumorigenesis. We demonstrated that genetic deletion or pharmacological inhibition of Syk using R788, skews macrophages in a pro-inflammatory state that leads to further changes in the TME by recruitment of CD8+ T cells and increased cytotoxicity against neuroblastoma tumor cells. Furthermore, combination treatment of R788 and anti-PDL1 mAb leads to complete tumor regression in 50% mice with durable anti-tumor immunity in small NB9464 tumors but not larger NB9464 tumors. However, combining radiation with R788 and anti-PDL1 mAb extended the survival of mice bearing large NB9464 tumors. Taken together, these data support Syk inhibition alone and in concert with anti-PDL1 mAb and radiation as a novel therapeutic strategy in neuroblastoma.

Our present analysis of the TME in human patient samples and murine NB models revealed an immunosuppressive TME with decreased infiltration of CD8+ T cells and increased infiltration of immunosuppressive TAMs in late-stage tumors. This data is in line with recent sc-RNA seq analysis performed on human NB patient samples which shows that Mono 2 (monocytes with high expression of genes related to antigen presentation and immunostimulation) was associated with better survival in high-risk neuroblastoma (15). However, Macro 1 (macrophages with immunosuppressive M2 gene signature) was significantly correlated with decreased survival. Furthermore, previous studies have shown that targeting immunosuppressive macrophages by using anti-CSF1R antibodies (19, 25) or stimulating the effector macrophages by using anti-CD40 antibodies (23, 24) have significantly decreased neuroblastoma growth in MYCN driven NB mouse models, suggesting that “re-educating” TAMs into immunostimulatory phenotype can be an effective strategy to improve responses to immunotherapy in NB.

A recent study found that Syk inhibition decreases cell viability of NB tumor cells that express Syk and potentiates cytotoxicity of chemotherapy drugs *in vitro* (40). In contrast to this study, we found that Syk is not expressed by human and murine NB cell lines, and Syk inhibition has no or modest effect on the viability of these cells. We found that macrophage-Syk is sufficient to promote NB growth and immunosuppression.

Syk is also reported to be present in dendritic cells and has a role in priming cytotoxic T-cell responses (61, 62). While, we have not tested the impact of Syk inhibition on DC’s capacity to generate CD8+ T cell responses, we found the predominant role of macrophage Syk in controlling tumor growth and inhibition of adaptive immune responses in the neuroblastoma model. We further confirm that macrophage Syk controls HIF1α stabilization to promote tumor growth and immunosuppression. HIF1α is previously reported to be involved in immunosuppressive macrophage differentiation and suppression of T cell function (63). Hence, studies combining R788 with VEGF inhibitors are warranted in the near future.

While immune checkpoint inhibitors, such as PD1 or PDL1, have been shown to improve outcomes in various adult tumors, their efficacy in pediatric tumors has thus far been limited (11, 64). Several studies have shown that PDL1 is expressed in NB tumors and correlates with worse outcomes (65, 66). Interestingly, a recent study has shown that chemotherapy significantly increased expression of PDL1 in NB tumors and specifically increased the expression of PDL1 in TAMs (54). We also found that TAMs from NB9464 tumors highly express PDL1 and R788 treatment further increased the expression of PDL1 in TAMs.

Studies have also found that treatment with anti-PDL1 and/or PD-1 antibodies in concert with anti-CD4 antibody, anti-CTLA4 mAb, anti-GD2 antibody, or a CSF-R1 inhibitor can significantly decrease tumor growth compared to monotherapy alone in neuroblastoma models *in vivo* (19, 54, 67, 68). We found that combination treatment with R788 and anti-PDL1 antibody results in complete tumor regression with durable anti-tumor immunity with rechallenge experiments *in vivo*. However, this treatment regimen was ineffective in mice bearing large NB9464 tumors. In support of this data, a recent study has shown that treatment of small NB9464-GD2 tumors (50 mm^3^) with extensive treatment regimen including radiation, hu14.18-IL2 immunocytokine (IC), anti-CD40, CpG, anti-CTLA-4 cured the mice but the treatment of large tumors (100 mm^3^) with this regimen only prolonged the survival with no mice cured (22, 26). However, we found that using R788 with radiation and anti-PDL1 mAb was effective in prolonging the survival of mice bearing NB9464 tumors even when started treatment at a later stage. Radiation may be functioning here to cytoreduce tumor volume as well as modulate the residual TME. Future studies will aim to identify other immunotherapeutic combinations that can cure mice with large tumors.

In conclusion, our results provide evidence that Syk is a marker of NB-associated macrophages, and therapeutic targeting of Syk with R788 reshapes the TME and sensitizes NB tumors to anti-PDL1 mAb. The data presented in this manuscript suggest that FDA-approved Syk inhibitor, fostamatinib, in combination with anti-PDL1 mAb and radiation, may be an effective strategy for NB and should be further explored in clinical investigations.

## Data availability statement

Data are available on reasonable request. The sequencing data and processed expression matrix have been deposited at the Gene Expression Omnibus with access number GSE224020, GSE224021, GSE224024, GSE224025. The code is avaialble on GitHub and can be accessed at: https://github.com/tpham2654/Neuroblastoma-SYK-RNAseq. The full list of differentially expressed genes for each experiment is available in **Supplementary Table S2**.

## Ethics statement

The use of human tissues was approved under the auspices of UCSD Institutional Review (Protocol 071729). Informed consent was obtained from all the patients. The deidentified biological samples were used under “exempt category 4” for research. All procedures involving animals were approved by the Institutional Animal Care and Use Committee of the University of California San Diego (Protocol S09398).

## Author contributions

SJ and DR conceived the idea and designed the study. DR, IP, TP, ET, and RJ performed experiments, analyzed, and interpreted data. IP and ET assisted in animal studies, including drug administration and tumor measurements. TP and PT assisted with RNA-seq data analysis and statistical analyses. RJ and AS assisted in designing radiation experiments. SJ, AS, AY, KL, and PT participated in the discussion of the data and contributed to the writing of the manuscript. All authors approved the final manuscript. SJ supervised the entire study and is the guarantor of this publication. All authors contributed to the article and approved the submitted version.
