# Supplementary material for: Targeting macrophage Syk enhances responses to immune checkpoint blockade and radiotherapy in high-risk neuroblastoma
Source: Front Immunol. 2023 Jun 7;14:1148317. doi: 10.3389/fimmu.2023.1148317 (PMC10283071; doi:10.3389/fimmu.2023.1148317)
Supplement: Supplementary file 1 [file DataSheet_1.pdf]

Supplementary Data

Legends to Supplementary Figures

Fig. S1: Gating strategy for the quantification of macrophages, B cells, and T cells. Total macrophages were evaluated using CD11b, Gr1, and F4/80 staining. Immunostimulatory macrophages were identified based on MHC II staining. B cells were evaluated using CD19 staining. T cells were evaluated using CD3, CD4, and CD8 staining. Effector T cells were evaluated using CD44 and CD62 staining.

Fig. S2: Immune composition of early-stage and late-stage MYCN amplified murine neuroblastoma tumors. FACS plots showing intratumoral CD11b+ myeloid cells, CD11b+Gr1- monocytes and CD11b+Gr1+ granulocytes, CD3+ T cells, CD4+ T cells, and CD8+ T cells in early-stage and late-stage murine NB9464 tumors.

Fig. S3: Macrophages foster NB progression: **A**. NB9464 cells were subcutaneously implanted in C57BL/6 WT mice (n = 5 mice/ gp) and treated with 50mg/kg anti-CSF1R antibody (administered intraperitoneally, every alternate day, until tumors were harvested) or ULTRA-LEAF^{TM} purified mAb (SA271G2) (250µg, administered once on day 10). Fig. shows the tumor volume of NB9464 tumors treated with anti-CSF1R ab or B cell depleting mAb (SA271G2). **B.** FACS quantification of CD11b+ F4/80 + macrophages in NB9464 tumors treated with anti-CSF1R mAb. **C.** FACS quantification of CD19+ B cells in NB9464 tumors treated with anti-CD20 mAb.

Fig. S4: Syk inhibition has no effect on the viability of NB cell lines: Cell viability assay showing no effect of Syk inhibitor R788 on the proliferation of SKNBE2 (A), IMR32 (B), and NB9464 (C) cells.

Fig. S5: Syk inhibition has no impact on T cell proliferation *ex vivo*: A-B. T cell proliferation *ex vivo* in CD90+T cells isolated from Syk^{MC-WT} and Syk^{MC-KO} mice or C57BL/6 WT mice and treated *ex vivo* with 500 nM R788.

Fig. S6. Gating strategy for the quantification of PDL1 + TAMs and tumor cells.

Table S1: List of differentially expressed genes in Syk^{MC-WT} and Syk^{MC-KO} TAMs, BMDMs stimulated with TCM media and treated with 500 nM R788, Vehicle NB9464 tumors, R788+ anti-PDL1-treated tumors and Radiation + R788 + anti-PDL1-treated NB9464 tumors.

Table S2: Patient characteristics for the samples used for immunohistochemistry studies.

References:

1. Dobin, A., et al., *STAR: ultrafast universal RNA-seq aligner.* Bioinformatics, 2013. **29**(1): p. 15-21.
2. Heinz, S., et al., Simple combinations of lineage-determining transcription factors prime cis-regulatory elements required for macrophage and B cell identities. Mol Cell, 2010. **38**(4): p. 576-89.
3. Dean, C.B. and J.D. Nielsen, Generalized linear mixed models: a review and some extensions. Lifetime Data Anal, 2007. **13**(4): p. 497-512.
4. Ritchie, M.E., et al., limma powers differential expression analyses for RNA-sequencing and microarray studies. Nucleic Acids Res, 2015. **43**(7): p. e47.
5. Kolde, R., et al., Host genetic variation and its microbiome interactions within the Human Microbiome Project. Genome Med, 2018. **10**(1): p. 6.

Fig. S1

A

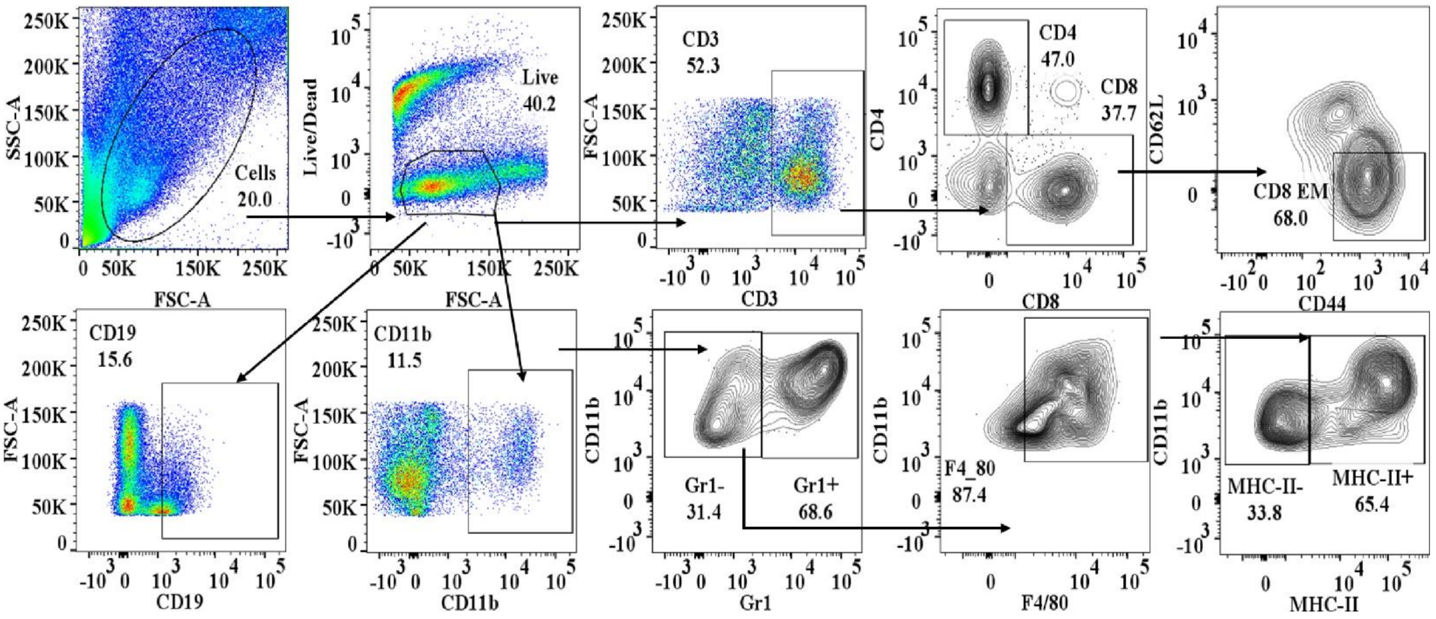

Cells
20.0
Live
40.2
CD3
52.3
CD4
47.0
CD8
37.7
CD8 EM
68.0
CD19
15.6
CD11b
11.5
Gr1-
31.4
Gr1+
68.6
F4_80
87.4
MHC-II-
33.8
MHC-II+
65.4
SSC-A
FSC-A
Live/Dead
CD3
CD4
CD8
CD62L
CD44
CD19
CD11b
Gr1
F4/80
MHC-II

Fig. S2

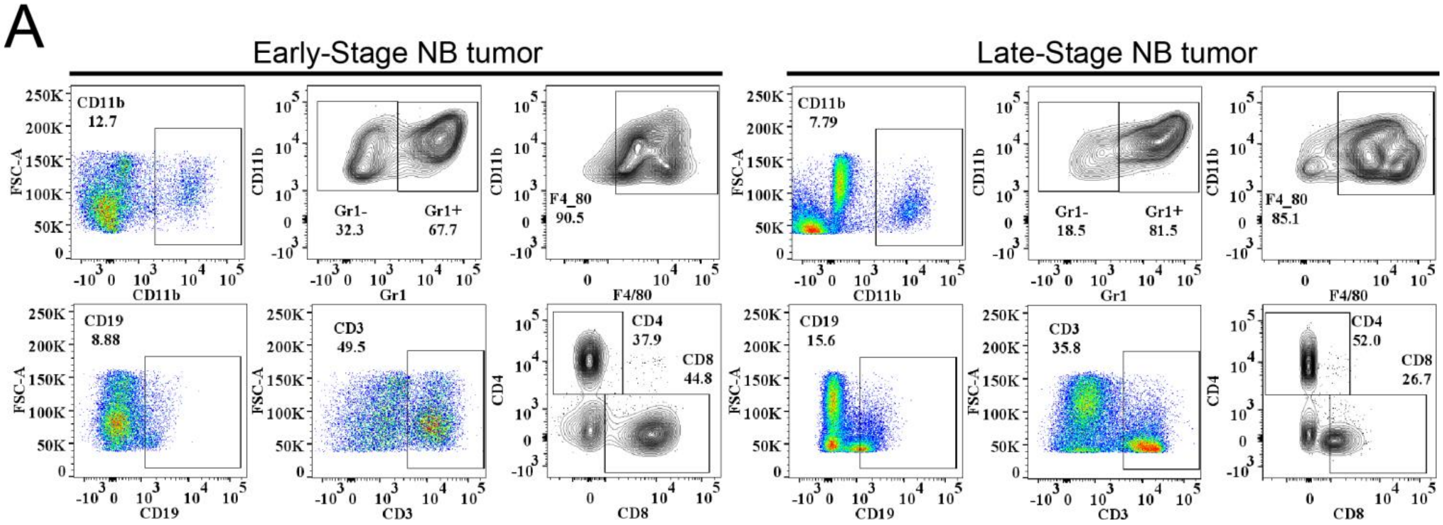

A
Early-Stage NB tumor
Late-Stage NB tumor
CD11b
12.7
Gr1-
32.3
Gr1+
67.7
F4_80
90.5
CD19
8.88
CD3
49.5
CD4
37.9
CD8
44.8
CD11b
7.79
Gr1-
18.5
Gr1+
81.5
F4_80
85.1
CD19
15.6
CD3
35.8
CD4
52.0
CD8
26.7
FSC-A
CD11b
Gr1
F4/80
CD19
CD3
CD4
CD8

Fig. S3

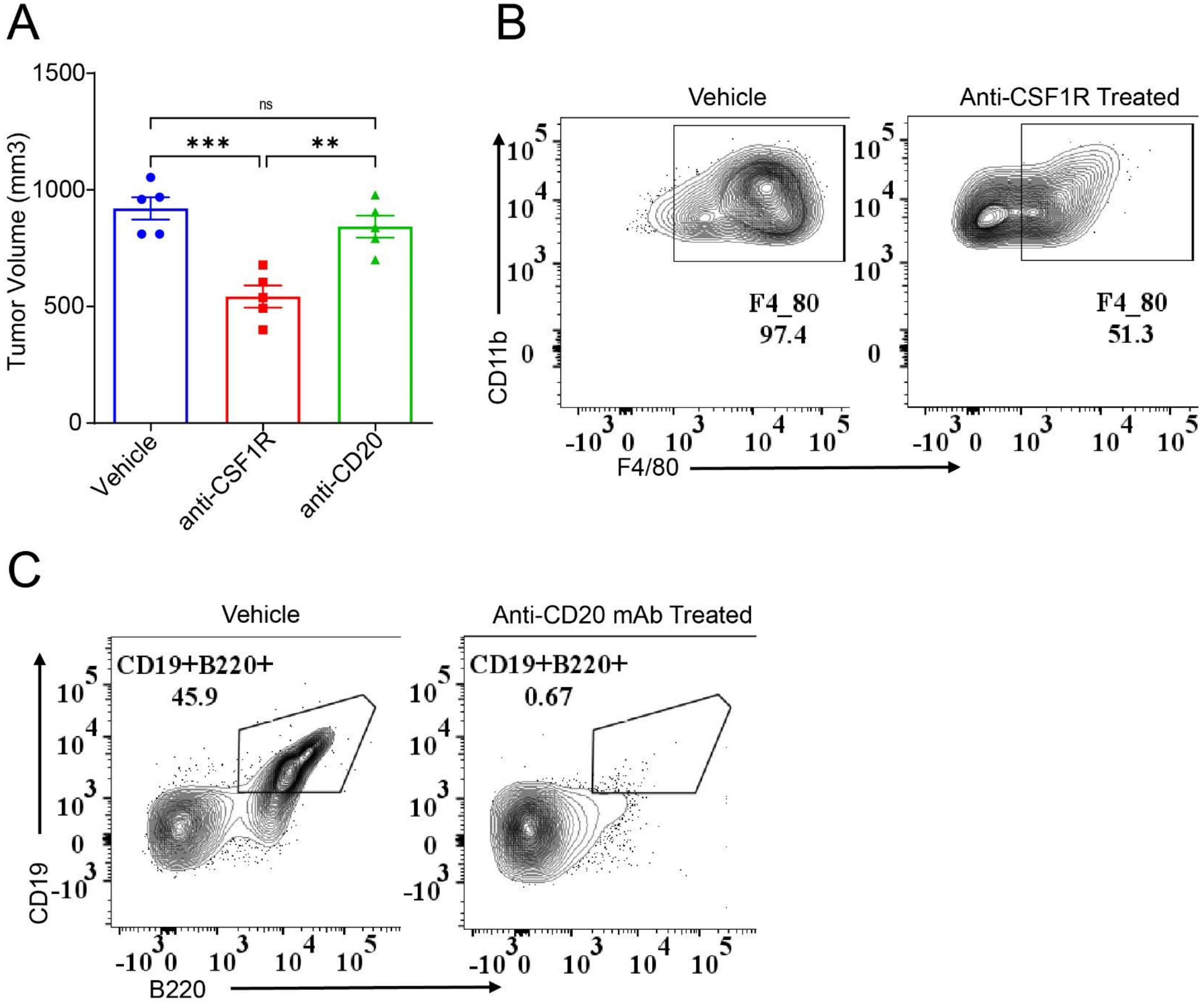

A
B
C
1500
1000
500
0
Tumor Volume (mm3)
ns
***
**
Vehicle
anti-CSF1R
anti-CD20
Vehicle
Anti-CSF1R Treated
F4_80
97.4
F4_80
51.3
CD11b
F4/80
Vehicle
Anti-CD20 mAb Treated
CD19+B220+
45.9
CD19+B220+
0.67
CD19
B220

Fig. S4

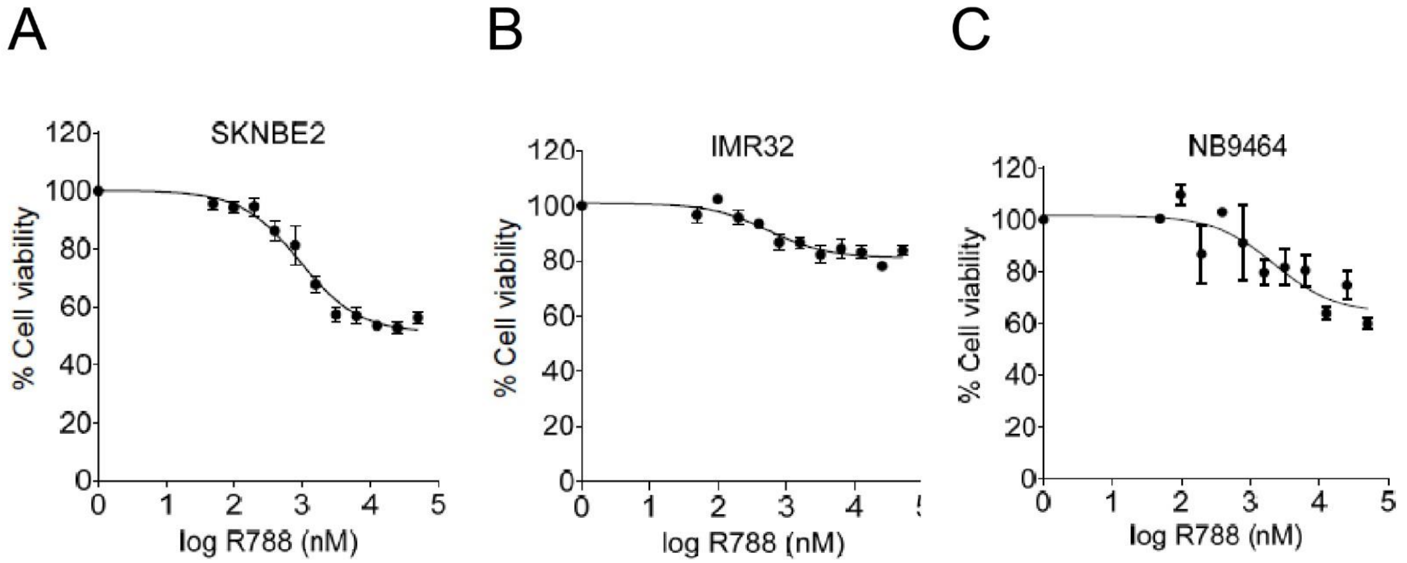
A
B
C
SKNBE2
IMR32
NB9464
% Cell viability
log R788 (nM)
0
20
40
60
80
100
120
1
2
3
4
5

Fig. S5

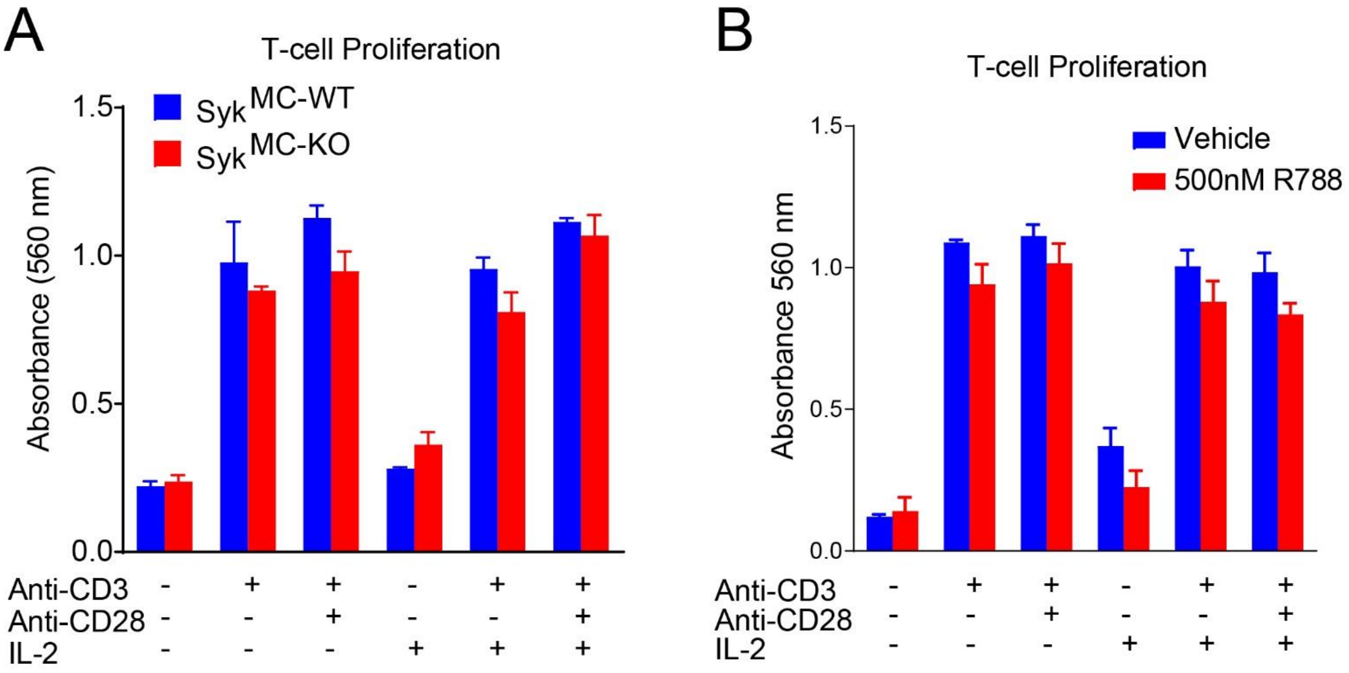
A
T-cell Proliferation
Syk MC-WT
Syk MC-KO
Absorbance (560 nm)
1.5
1.0
0.5
0.0
Anti-CD3 - + + - + +
Anti-CD28 - - + - - +
IL-2 - - - + + +
B
T-cell Proliferation
Vehicle
500nM R788
Absorbance 560 nm
1.5
1.0
0.5
0.0
Anti-CD3 - + + - + +
Anti-CD28 - - + - - +
IL-2 - - - + + +

Fig. S6

A

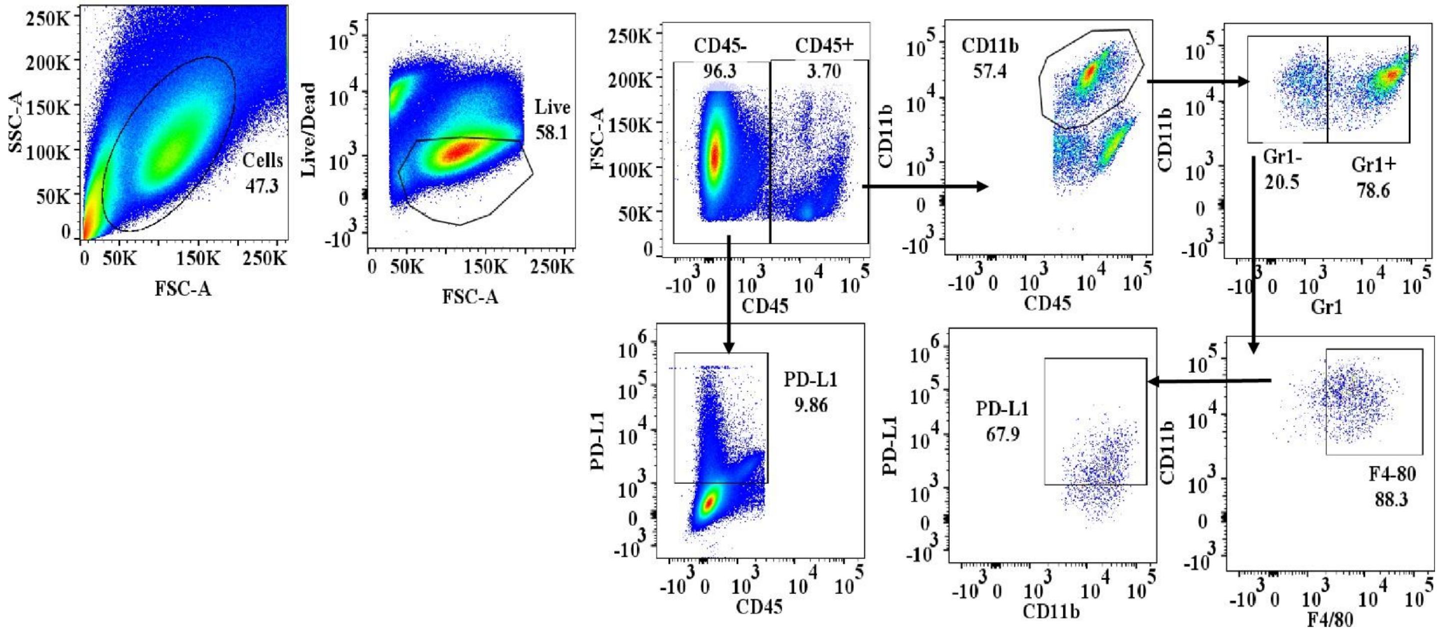
SSC-A
FSC-A
Cells
47.3
Live/Dead
Live
58.1
CD45-
96.3
CD45+
3.70
CD11b
57.4
Gr1-
20.5
Gr1+
78.6
PD-L1
9.86
PD-L1
67.9
F4-80
88.3
CD45
CD11b
Gr1
F4/80
